# A study of sertraline in dialysis (ASSertID): a protocol for a pilot randomised controlled trial of drug treatment for depression in patients undergoing haemodialysis

**DOI:** 10.1186/s12882-015-0170-x

**Published:** 2015-10-26

**Authors:** Karin Friedli, Michael Almond, Clara Day, Joseph Chilcot, Maria da Silva Gane, Andrew Davenport, Ayman Guirguis, Naomi Fineberg, Benjamin Spencer, David Wellsted, Ken Farrington

**Affiliations:** Centre for Lifespan and Chronic Illness Research, Department of Psychology, School of Life and Medical Sciences, University of Hertfordshire, College Lane Campus, Hatfield, AL10 9AB UK; Renal Unit, Lister Hospital, East & North Herts NHS Trust, Coreys Mill Lane, Stevenage, SG1 4AB UK; Department of Renal Medicine, University Hospitals Birmingham NHS Foundation Trust, Queen Elizabeth Hospital, Queen Elizabeth Medical Centre, Birmingham, B15 2TH UK; Southend University Hospital NHS Foundation Trust, Prittlewell Chase, Westcliff – On – Sea, Essex, SSO ORY, UK; University College London Centre for Nephrology, Royal Free Hampstead NHS Trust, Rowland Hill Street, London, NW3 2PF UK; Health Psychology Section, Psychology Department, Institute of Psychiatry, Psychology and Neuroscience, King’s College London, 5th floor Bermondsey Wing, Guy’s Campus, London Bridge, London, SE1 9RT UK; Hertfordshire Partnership University NHS Foundation Trust, Rosanne House, Welwyn Garden City, AL8 6HG UK; Department of Psychological Medicine, Institute of Psychiatry, Psychology and Neuroscience, King’s College London, Weston Education Centre, 10 Cutcombe Street, London, SE5 9RJ UK; South London and Maudsley NHS Foundation Trust, Maudsley Hospital, Denmark Hill, London, SE5 8AZ UK; Postgraduate Medical School, University of Hertfordshire, College Lane Campus, Hatfield, AL10 9AB UK

**Keywords:** Depression, End stage renal disease, Haemodialysis, Sertraline, Feasibility RCT

## Abstract

**Background:**

The prevalence of depression in people receiving haemodialysis is high with estimates varying between 20 and 40 %. There is little research on the effectiveness of antidepressants in dialysis patients with the few clinical trials suffering significant methodological issues. We plan to carry out a study to evaluate the feasibility of conducting a randomised controlled trial in patients on haemodialysis who have diagnosed Major Depressive Disorder.

**Methods/Design:**

The study has two phases, a screening phase and the randomised controlled trial. Patients will be screened initially with the Beck Depression Inventory to estimate the number of patients who score 16 or above. These patients will be invited to an interview with a psychiatrist who will invite those with a diagnosis of Major Depressive Disorder to take part in the trial. Consenting patients will be randomised to either Sertraline or placebo. Patients will be followed-up for 6 months.

Demographic and clinical data will be collected at screening interview, baseline interview and 2 weeks, and every month (up to 6 months) after baseline. The primary outcome is to evaluate the feasibility of conducting a randomised, double blind, placebo pilot trial in haemodialysis patients with depression. Secondary outcomes include estimation of the variability in the outcome measures for the treatment and placebo arms, which will allow for a future adequately powered definitive trial. Analysis will primarily be descriptive, including the number of patients eligible for the trial, drug exposure of Sertraline in haemodialysis patients and the patient experience of participating in this trial.

**Discussion:**

There is an urgent need for this research in the dialysis population because of the dearth of good quality and adequately powered studies. Research with renal patients is particularly difficult as they often have complex medical needs. This research will therefore not only assess the outcome of anti-depressants in haemodialysis patients with depression but also the process of running a randomised controlled trial in this population. Hence, the outputs of this feasibility study will be used to inform the design and methodology of a definitive study, adequately powered to determine the efficacy of anti-depressants in patient on haemodialysis with depression.

**Trial registration:**

ISRCTN registry ISRCTN06146268 and EudraCT reference: 2012-000547-27.

## Background

The prevalence of people receiving renal replacement therapy (RRT) for end stage renal disease (ESRD) is increasing worldwide. In the UK around 900 people per million were receiving these therapies in 2013, with approximately equal numbers on dialysis and transplantation [1]. These numbers continue to increase by about 4 % annually. People on dialysis have a high symptom burden and a greatly increased mortality [2, 3]. Depression is common but difficult to diagnose because of the symptom overlap between depression and advanced kidney disease [4]. Estimates of prevalence of depression in this population vary from around 40 %, based on self-reported questionnaire screening, to around 20 %, on psychiatric interview [4, 5]. Depression in dialysis patients is associated with reduced quality of life, increased prevalence of cardiovascular disease, and increased mortality [6, 7]. Depression may also lead to reduced treatment adherence, reduced self-care behaviour, and subsequently greater healthcare resource utilisation [8, 9]. Therefore attempts to identify feasible and effective treatments for depression in this setting remain a clinical priority.

There has been little research on the effectiveness of antidepressant medication in dialysis patients. A 2009 Cochrane review identified only one Randomised Controlled Trial (RCT), a small trial with 14 patients, which had inconclusive results [10, 11]. While the trends indicated that Fluoxetine was more effective than placebo, the study was under powered. Other studies undertaken to date have similarly been of limited size and design and have lacked appropriate control groups [12–16]. It is perhaps unsurprising that a recent systematic review, including recommendations by the European Renal Best Practice Group, recommended a well-designed RCT in this setting [17].

In keeping with this recommendation, our primary outcome is to undertake a study to evaluate the feasibility of conducting a randomised, double blind, placebo controlled trial in patients with ESRD on haemodialysis (HD) who have a diagnosis of Major Depressive Disorder (MDD) according to DSM-IV. Our study will explore key methodological, design, safety and drug exposure and acceptability issues, including the number of ESRD patients who are eligible to take part in the trial, in order to facilitate the design of a subsequent large scale study. Our secondary aims are to estimate the variability in the outcome measures for the treatment and placebo arms, allowing an assessment of effect sizes, effects of treatment centre, and bias due to missing data in order to power a future definitive trial in this setting. The antidepressant under investigation will be Sertraline, a licensed selective serotonin reuptake inhibitor (SSRI). Of the SSRIs available a recent meta-analysis recommended Sertraline due to its favourable balance between efficacy and acceptability, and low cost [18]. Sertraline also has a robust safety profile for cardiac disease [19], the prevalence of which is high in ESRD. It is also highly protein bound and almost entirely hepatically cleared [20]. No dose adjustment is believed to be required in ESRD though there is some evidence that Sertraline’s elimination half-life is extended in ESRD [21]. Thus our study will also determine pre- and post-dialysis drug levels in these haemodialysed patients.

## Methods/Design

### Funding and governance

The study is funded by the National Institute for Health Research programme, Research for Patient Benefit. The reference number is PB-PG-0110-21073. The study received ethical approval from NRES London-Harrow (REC reference 12/LO/1554; IRAS project ID 100774). The trial is co-sponsored by East and North Herts Trust NHS Trust and the University of Hertfordshire. The Clinical Trials Unit involved is Norwich Clinical Trials Unit, University of East Anglia. The conduct of the trial will be overseen by a Steering group which will meet at least 6 monthly and will include the Chief Investigator (CI), all Principal Investigators (PIs), the Trial manager and lay members. A Data Monitoring and Ethics Committee, consisting of independent statistician, clinician, and lay person, will meet at least twice during the course of the study.

### Setting

The study will take place in four UK Renal Centres, the Lister Hospital in Stevenage, Hertfordshire, Southend Hospital in Essex, the Royal Free Hospital in London, and the Queen Elizabeth Hospital in Birmingham. By the end of 2012, the number of adults receiving HD at each centre was as follows: Lister Hospital 409 patients, Southend 118 patients, Royal Free Hospital 714 patients and Queen Elizabeth Hospital 926 patients [22].

### Overview of design

The study is planned in two phases – a screening phase and a trial phase.

#### Screening phase

Initially patients on HD will be screened for eligibility using the Beck Depression Inventory, version II (BDI-II) [23] and other questionnaires. Patients who have been identified on screening to have a high likelihood of suffering from MDD, and who meet the eligibility criteria will be invited to see the study psychiatrist to carry out a diagnostic interview [24, 25]. Those diagnosed with MDD will be invited to participate in the randomised controlled trial.

#### Trial phase

**Fig. 1:**
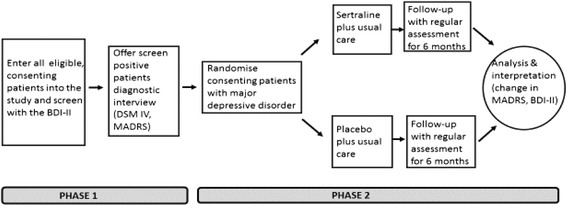
Schematic diagram of overall design

Consenting patients with be randomised to receive either sertraline, initial dose 50 mg daily, or placebo for a planned duration of 6 months with follow up at 2 weeks and then monthly (Fig. 1)

### Participants

#### Inclusion/Exclusion criteria

##### Screening

Patients with ESRD, aged 18 or over, who have been on HD for at least 3 months, and who speak and read English well enough to complete the questionnaires.

##### Psychiatric interview

Patients with a BDI-II [23] of 16 or more on screening with a likely prognosis of more than one year. Exclusion criteria include: treatment for anxiety or depression during the previous 3 months with either antidepressants or formal psychological therapy; planned living donor transplant within the period of the trial; pregnancy or childbearing potential without adequate birth control; contraindicated coexistent drug therapy (sertraline SmPC), including triptans, antipsychotics, dopamine antagonists, tramadol, linezolid, warfarin; hepatic impairment - alanine transaminase more than twice the upper limit of normal and/or INR greater than 1.3; Hepatitis, HIV/AIDS, and Creutzfeldt-Jakob disease.

##### Trial phase

Patients with a diagnosis of mild to moderate MDD according to a DSM-IV interview, who score 18 or above on the MADRS [25], and who have the capacity to understand the trial and to give consent. Exclusions will include: a diagnosis of a severe MDD and those judged to be at moderate to severe risk of self-harm who will be referred immediately for further psychiatric evaluation; other psychiatric conditions including substance dependency, psychosis, personality disorder, dementia or panic disorder, with the exception of other anxiety disorders.

### Sample size

A sample size of 60 in the Trial Phase will provide estimates of the population variance to a precision of 1.2 x the sample variance allowing reliable estimates to be derived for the outcome measures, and inform design of the planned full scale trial. This will require 800 patients to be screened. The assumptions on which this estimate is based are: 30 % of patients will screen positive on the BDI-II [22] and that approximately 50 % of those patients will be subsequently be diagnosed with MDD following a psychiatric interview and that 50 % will agree to take part on the Trial Outcomes phase – e.g. 7.5 % of the sampled patients.

### Consent

Subjects will be required to give informed consent up to four occasions, the screening phase, for psychiatric interview, for the trial phase, and for a qualitative interview conducted towards the end of the trial phase.

### Randomisation

Following the diagnostic interview, eligible, consenting patients with diagnosed MDD will be randomised by the research psychiatrist. Both the research psychiatrist and patient will be blind to the allocation. Randomisation will take place in blocks using pre-prepared codes for each centre. These will be incorporated into a protected web based randomisation programme prepared by Norwich CTU. Only the research psychiatrist will have authorised access to the online randomisation programme. Following randomisation the relevant pharmacy will be informed of the allocation (treatment A or B) by automatically generated email. The pharmacist will be blind to the allocation. The CTU will hold the patient-specific allocation data on a secure server. The CI and PI at each centre will have access to this data file only via a special log-in should the need arise to unblind. No user identifiable data will stored in the randomisation database. Web traffic will be encrypted using standard secure sockets layer technology.

### Intervention and control group

#### Intervention group

The drug under investigation is Sertraline hydrochloride which will be provided in over-encapsulated form identical to the placebo.

#### Control group

The study placebo is microcrystalline cellulose and magnesium stearate. The placebo has the identical over encapsulation as the Sertraline tablets.

#### Administration, dosage and dosage regime on study medication

After randomisation, patients will be prescribed one capsule daily of Sertraline hydrochloride (50 mg) or an identical capsule of placebo. Patients will be reviewed after two weeks by the research psychiatrist or another mental health professional to assess mental state and tolerance of the drug. Dose escalation to a maximum of 100 mg/day will be allowed if indicated on further assessment by the research psychiatrist at 2 and 4 months.

### Data collection

Data will be collected at the six time points: screening interview, baseline, 2 weeks, 2, 4 and 6 months after baseline. Patients will be seen at baseline and monthly during follow-up by the research nurse and by the study psychiatrist at baseline, 2 weeks and 2, 4 and 6 months (see Table 1).

**Table 1:** Study assessments

	Screening	Psychiatric interview	Entry to clinical trial	1-2 weeks	1 month	2 months	3 months	4 months	5 months	6 months
Informed Consent	X	X	X							
Inclusion/exclusion criteria fulfilled	X	X	X							
Demographics	X									
Co-morbid conditions (self-report)	X									
Charlson Co-morbidity Index [29]			X		X	X	X	X	X	X
Brief psychiatric history	X									
BDI-II [23]	X									X
PHQ-9 [26]	X			X	X	X	X	X	X	X
P4 Suicidality Screener [33]				X	X		X		X	
Psychiatric Assessment		X		X		X		X		X
Montgomery- Asberg Depression Rating Scale [25]		X				X		X		X
Mini International Neuropsychiatric Interview [24]		X								
List of medications			X		X	X	X	X	X	X
Description of dialysis treatment			X		X	X	X	X	X	X
Mid-week pre and post dialysis blood pressure	X		X		X	X	X	X	X	X
Dry weight	X		X		X	X	X	X	X	X
Adherence to dialysis treatment	X		X		X	X	X	X	X	X
Interdialytic weight gain	X		X		X	X	X	X	X	X
Urine volume per 24 h	X									
Dialysis adequacy Kt/V	X		X		X	X	X	X	X	X
Dialysis time	X		X		X	X	X	X	X	X
Urea & electrolytes^a^	X		X		X	X	X	X	X	X
Full blood count^b^	X		X		X	X	X	X	X	X
Liver function tests^c^			X		X	X	X	X	X	X
Electrocardiogram			X			X		X		X
IMP review					X	X	X	X	X	X
Drug compliance				X	X	X	X	X	X	X
Sertraline Plasma blood test								X		
Baseline assessment of signs and symptoms			X							
Adverse events				X	X	X	X	X	X	X
Kidney Disease QoL questionnaire [30]			X			X		X		X
Euroqol EQ 5D questionnaire [22, 31]			X			X		X		X
Clinical Global Impression Severity Scale [28]		X				X		X		X
Clinical Global Impression Improvement Scale [28]						X		X		X

^a^Urea, Creatinine, Calcium, Sodium, Potassium and Bicarbonate

^b^Haemoglobin, White blood cell count and platelets

^c^Bilirubin, Albumin, Alanine transaminase (ALT), Aspartate transaminase (AST), Aspartate Aminotransferase, Calcium, Phosphate and C-Reactive Protein

#### Screening

1. Socio-demographic data; primary renal disease; RRT history; history of depression and other psychological/psychiatric illness; non-renal comorbidity; baseline haematological and biochemical parameters including dialysis adequacy.
2. Questionnaire data – BDI-II [23]; Patient Health Questionnaire-9 (PHQ-9) [26].

#### Psychiatric interview

Mini-International Neuropsychiatric Interview (MINI) version 6.0 [24]; Folstein Mini-Mental Status Exam [27]; Montgomery-Asberg Depression Rating Scale [25] (MADRS), Clinical Global Impression – Severity Scale [28].

The research psychiatrist will also assess each patient for suicide risk, clinically and with reference to item 10 on the MADRS and question A3G on the MINI. Patients at significant risk will be excluded from the study, and referred for further urgent psychiatric assessment.

#### Trial phase

##### Baseline

Charlson Co-morbidity Index [29]; medications; electrocardiogram; routine haematological and biochemical parameters, dialysis parameters (see table 1), BDI-II [23], PHQ-9 [26], MADRS [25], KD-QoL [30] and EQ-5D [22, 31].

##### Follow-up

Clinical events; changes in medication, routine biochemical and haematological parameters, dialysis parameters, PHQ-9 (all monthly) [26], MADRS [25], Clinical Global Impression – Severity Scale [28], Clinical Global Impression - Improvement Scale [28], KDQoL [30] and EQ-5D (2, 4 and 6 months) [22, 31].

##### Other assessments during follow-up

1. A semi-structured interview enquiring about patients experiences during the study eg burden of questionnaires and additional medication, views on information leaflets.
2. Pre- and post-dialysis blood samples for subsequent analysis of sertraline levels carried out on one occasion between 3 and 5 months.

### Adverse events (AEs) and serious adverse events (SAEs)

Adverse events will be recorded on the case report forms. Particular attention will be paid to mood deterioration which will, if significant or involving increase suicide risk, trigger review by study psychiatrist. SAEs will be reported to the CI and sponsors within 24 h. Events which result in death, threat to life, disability, hospitalisation, or congenital abnormality or birth defect will constitute SAEs. Hospitalisation for planned procedures including access surgery will not be regarded as SAEs.

### Emergency unblinding

Blinding can be broken in a medical emergency where the knowledge of the blinded treatment is necessary such as deterioration in mood involving suicidal thoughts, or attempted suicide, cardiac dysrhythmias, GI bleed, suspected serotonin syndrome or neuroleptic malignant syndrome, accidental overdose eg by a child in a participants household, and in the event of a Suspected Unexpected Serious Adverse Reaction (SUSAR).

### Data handling and management

All data collected will be recorded on paper CRFs or the standardised questionnaires and subsequently entered by the research staff at each site on to the password-protected electronic web-based database controlled by the Data Manager at the Norwich CTU. Regular reports will be produced including accrual rates and patient progress though the study, and missing data.

### Data analysis

The primary outcome is to evaluate the feasibility of conducting a randomised, double blind, placebo pilot trial in ESRD patients with MDD. This includes the number of ESRD patients eligible for this clinical trial, safety and drug exposure of Sertraline in ESRD patients and the patient experience of participating in this trial. The secondary outcomes are to estimate the variability in the outcome measures for the treatment and placebo arms, allowing an assessment of effect sizes, effects of treatment centre, and bias due to missing data. The study will also try to evaluate the gain in the outcome measures in treated patients compared to those in the control group and whether changes in these measures over time can be attributed to treatment. This is a pilot study and a statistically significant outcome in this domain is unlikely. Establishing the effect size though will be of crucial importance in powering future definitive trials.

#### Screening data

The primary purpose of the screening phase is to select patients with a BDI-II of 16 or more who meet the criteria to go forward for psychiatric interview to diagnose MDD. We will analyse the proportions of patients meeting the inclusion criteria; the proportion refusing to be screened; and the proportion of patients who screen positive. The characteristics of these groups will be defined as far as possible. Baseline characteristics for all patients randomised will be evaluated (means, proportions, counts) for patients in the treatment and placebo arms. To meet the CONSORT [32] reporting criteria, the flow of patients through recruitment to this phase of the study will be recorded, and the numbers of patients falling into each group evaluated.

#### Trial process data

The primary analysis will be descriptive, seeking to characterise the acceptability of the study to patients by estimating the proportions of patients who agree to take part in this phase and those withdrawing from randomised treatment. The degree of adherence to the randomised treatment will also be evaluated to inform future trial design. The analysis will also characterise the missing data, and seek to determine the extent to which bias is introduced via missing data. Lastly, the nature and number of the reported AEs will be classified. This will help to evaluate the safety profile of the study drug.

#### Variability of outcome measures

The aim of this analysis will be to characterise the variability of the outcome measures at 6 months, and the related effect size of treatment versus placebo for each outcome. Using an “as treated” sample the effect size (cohen’s d) will be estimated for all the outcome measures. Analysis will also consider the influence of covariates on the outcome measures to determine the need for stratification in the larger RCT to follow, and estimate bias introduced by missing data and non-completion. Further analysis will examine the relationship between quality of life and depression scores over time.

#### Drug exposure

Descriptive data on the levels of Sertraline will allow inferences on the effect of HD on drug removal.

## Discussion

There is a vital need for such a study both in dialysis and pre dialysis situation since evidence is lacking. We aim to increase our understanding of the complexities of conducting research in this population, not just in terms of outcome but also process. The outputs of this feasibility study will be used to inform the design and methodology of a definitive study, adequately powered to evaluate the efficacy of anti-depressants in ESRD patients on HD and with a MDD. It is likely this is the forerunner of a larger study of antidepressants in this population and this will be an important outcome of this research. We aim to disseminate the results of this study in the renal and psychiatric peer-reviewed scientific journals as well as conferences and the wider lay community.

## Abbreviations

AEs: Adverse Events
BDI-II: Beck Depression Inventory – Version II
CI: Chief Investigator
CRFs: Case Report Forms
CTU: Clinical Trials Unit
DSM-IV: Diagnostic and Statistical Manual of Mental Disorders, 4th Edition
EQ-5D: EuroQol - 5D
ESRD: End Stage Renal Disease
HD: Haemodialysis
IMP: Investigational Medicinal Product
KD-QoL: Kidney Disease Quality of Life instrument
Kt/V: Urea Clearance normalised to total body water
MADRS: Montgomery-Åsberg Depression Rating Scale
MDD: Major Depressive Disorder
MINI: Mini-International Neuropsychiatric Interview
NRES: National Research Ethics Service
PHQ-9: Patient Health Questionnaire 9
PI: Prinicipal Investigator
RCT: Randomised Controlled Trial
RRT: Renal Replacement Therapy
SAEs: Serious Adverse Events
SmPC: Summary of Product Characteristics
SSRI: Selective Serotonin Reuptake Inhibitor
SUSAR: Suspected Unexpected Serious Adverse Reaction
